# Hypotension Prediction Index software for management of hypotension during moderate- to high-risk noncardiac surgery: protocol for a randomized trial

**DOI:** 10.1186/s13063-019-3329-0

**Published:** 2019-05-03

**Authors:** Kamal Maheshwari, Tetsuya Shimada, Jonathan Fang, Ilker Ince, Edward J. Mascha, Alparslan Turan, Andrea Kurz, Daniel I. Sessler

**Affiliations:** 1 Department of Outcomes Research, Anesthesiology Institute, Cleveland Clinic, 9500 Euclid Avenue, E-31, Cleveland, OH 44195 USA; 2 Department of General Anesthesiology, Anesthesiology Institute, Cleveland Clinic, 9500 Euclid Avenue, E-31, Cleveland, OH 44195 USA; 3 Department of Anesthesiology, National Defense Medical College, Tokorozawa, Saitama Japan; 4 Department of Anesthesiology and Reanimation, Ataturk University School of Medicine, Erzurum, Turkey; 5 Departments of Quantitative Health Sciences and Outcomes Research, Lerner Research Institute and Anesthesiology Institute, Cleveland Clinic, Cleveland, OH USA

## Abstract

**Background:**

Hypotension is associated with serious complications, including myocardial infarction, acute kidney injury, and mortality. Consequently, predicting and preventing hypotension may improve outcomes. We will therefore determine if use of a novel hypotension prediction tool reduces the duration and severity of hypotension in patients having non-cardiac surgery.

**Methods/design:**

We will conduct a two-center, pragmatic, randomized controlled trial (*N* = 213) in noncardiac surgical patients > 45 years old who require intra-arterial blood pressure monitoring. All participating patients will be connected to a Flortrac IQ sensor and EV1000 platform (Edwards Lifesciences, Irvine). They will be randomly assigned to blinded or unblinded arms. The Hypotension Prediction Index (HPI) and advanced hemodynamic information will be universally recorded, but will only be available to clinicians when patients are assigned to unblinded monitoring. The primary outcome will be the effect of HPI software guidance on intraoperative time-weighted average mean arterial pressure under a threshold of 65 mmHg, which will be assessed with a Wilcoxon-Mann-Whitney 2-sample, two-tailed test.

**Discussion:**

Our trial will determine whether the Hypotension Prediction Index and associated hemodynamic information substantively reduces hypotension during non-cardiac surgery.

**Trial registration:**

ClinicalTrials.gov, NCT03610165. Registered on 1 August 2018.

**Electronic supplementary material:**

The online version of this article (10.1186/s13063-019-3329-0) contains supplementary material, which is available to authorized users.

## Background

Intraoperative hypotension is surprisingly common and, depending upon the definition, has an incidence ranging from 5 to 99% [1]. Most patients having non-cardiac surgery experience at least one episode during which mean arterial pressure (MAP) decreases to < 65 mmHg, often shortly after anesthetic induction [2, 3]. Intraoperative hypotension is associated with complications, including myocardial infarction, acute kidney injury, and mortality [4–7]. A recent randomized controlled trial reported that preventing intraoperative hypotension reduces the risk of postoperative organ dysfunction by about a quarter. This important result suggests that the association between hypotension and organ injury is at least partially causal and, therefore, amenable to intervention [8].

It seems likely that reducing the frequency, depth, and duration of intraoperative hypotension will reduce organ injury. Currently, anesthesia clinicians respond to blood pressure trends and treat hypotension as necessary when it occurs. But in practice, it is difficult to predict, and therefore prevent, intraoperative hypotension.

The Hypotension Prediction Index (HPI) is derived from arterial waveforms and predicts hypotension, and the software also provides advanced hemodynamic data including cardiac output, vascular elastance, and stroke volume—which presumably help clinicians select optimal treatments. HPI uses arterial waveform features, including waveform time, amplitude, area, segment slopes, and complexity features, to predict hypotension, defined by MAP < 65 mmHg for at least one minute [9]. The sensitivity and specificity of HPI for predicting hypotension 5 min beforehand is 92% and 92%; it is 89% and 90% 10 min beforehand and 88% and 87% 15 min beforehand [9]. HPI software is commercially available in Europe and the United States.

We propose to compare the amount of intraoperative hypotension below a MAP of 65 mmHg in patients requiring invasive arterial pressure monitoring who are randomized to blinded vs unblinded HPI monitoring. Specifically, we will test the primary hypothesis that adding HPI software guidance to the information provided by the invasive arterial pressure monitoring during moderate-to-high-risk noncardiac surgery reduces time-weighted average (TWA) intraoperative hypotension below a threshold of 65 mmHg. Secondarily, we will study the effect of HPI software guidance on TWA MAP below 60 and 55 mmHg.

## Methods/design

**Fig. 1:**
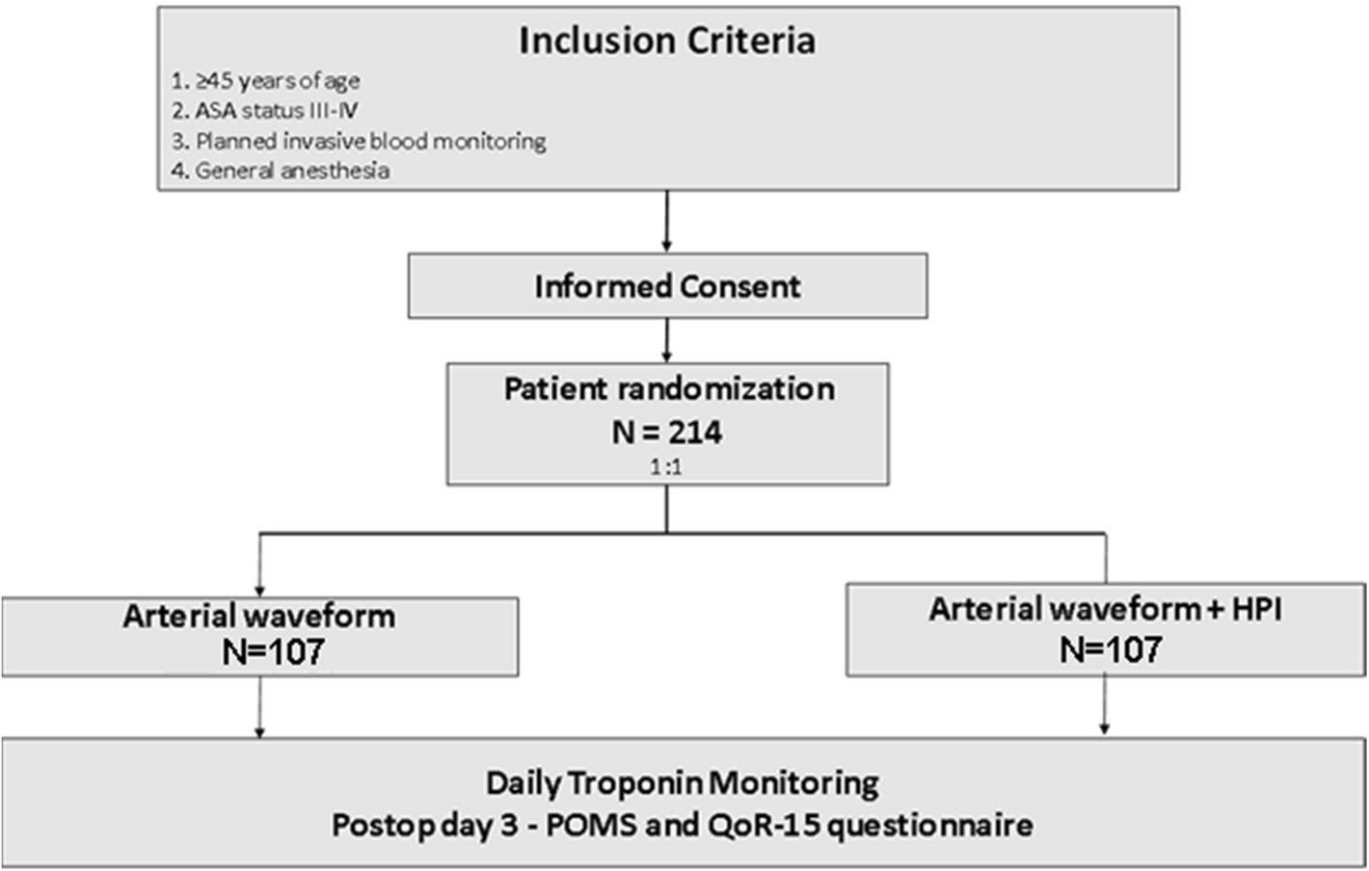
Study flow chart. *ASA* American Society of Anesthesiologists, *HPI* hypotension prediction index, *POMS* postoperative morbidity survey, *QoR-15* quality of recovery

We will conduct a trial comparing the amount of hypotension in patients requiring invasive arterial pressure monitoring and randomized to blinded vs unblinded HPI monitoring (Fig. 1). We followed SPIRIT recommendations for interventional trial (Addtional file 1). Informed consent will be obtained from all participating patients. The study sites will be the Cleveland Clinic Main Campus and the Cleveland Clinic Fairview Hospital. Ethics committee approval was obtained from the Cleveland Clinic Institutional Review Board. The trial was registered at ClinicalTrials.gov on July 13, 2018 (NCT03610165) under the principal investigator Kamal Maheshwari.

Written informed consent will be obtained from all included patients. Enrolled patients will be at least 45 years old, have an American Society of Anesthesiologists (ASA) physical status III–IV, be scheduled for elective major non-cardiac surgery with general anesthesia, be planned to have intra-arterial pressure monitoring, and have at least overnight hospitalization. Patients will be excluded if they are pregnant or have known clinically important intra-cardiac shunts, aortic stenosis with valve area ≤ 1.5 cm^2^, moderate to severe aortic and/or mitral regurgitation, and moderate to severe mitral stenosis. Patients will also be excluded if they have persistent atrial fibrillation, or congestive heart failure with ejection fraction < 35%. And finally, we will exclude patients scheduled for neurosurgery or cardiovascular procedures.

Potentially qualifying patients will be evaluated during their preoperative anesthesia clinic visits. Written informed consent will be obtained from each, and each will be informed that they may decline to participate or withdraw from the study at any time.

### Protocol

**Fig. 2:**
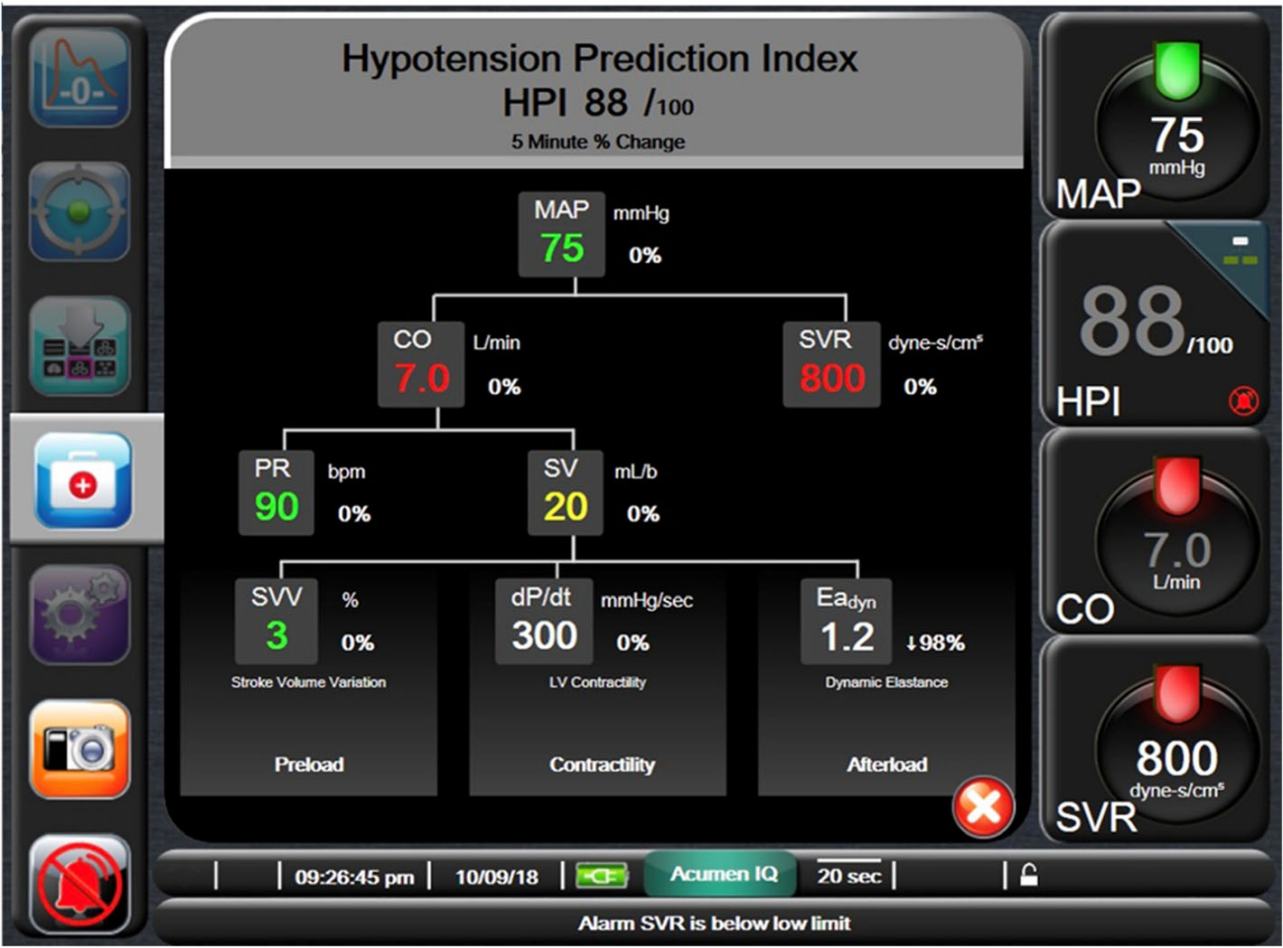
Advanced hemodynamic information. In the screenshot the Hypotension Prediction Index (*HPI*) is 88% and hemodynamic parameters mean arterial pressure (*MAP*), pulse rate (*PR*), cardiac output (*CO*), stroke volume (*SV*), stroke volume variation (*SVV*), rate of contractility (*dP/dt*), dynamic elastance (*Ea*_*dyn*_) are shown

There will be no restriction on type of general anesthesia, and regional anesthesia/analgesia will be permitted. In addition to the routine anesthetic monitoring, an arterial catheter will be inserted for direct pressure monitoring. The catheter will be connected to a FloTrac IQ sensor, and the sensor connected to an EV1000 platform which includes HPI software (Edwards Lifesciences, Irvine, CA; Fig. 2).

Shortly before case start, patients will be randomly assigned to blinded or unblinded HPI software monitoring by the investigator not involved in clinical care. Randomization will be stratified by study site, with randomly sized blocks achieving a 1:1 balance within site. Randomization codes will be reproducible, generated using the PROC procedure in SAS statistical software, and implemented via a web-based system (REDCap secure web application). Allocation will thus be concealed, and patients will not be informed of their group assignments. The randomized groups will be:

- **Blinded HPI software:** Arterial waveform and pressures displayed on GE CARESCAPE patient monitor.
- **Unblinded HPI software:** Arterial waveform and pressures displayed on GE CARESCAPE patient monitor and the Hypotension Prediction Index software-enabled EV1000 platform.

Anesthetic dose, the amount and timing of intravenous fluids and use of vasopressor and inotropic drugs will be determined by the anesthesia team and based on available information in both groups. For patients assigned to blinded monitoring, decisions will be based on patient and surgical characteristics, signals from all routine anesthesia monitors, and the arterial waveform.

**Fig. 3:**
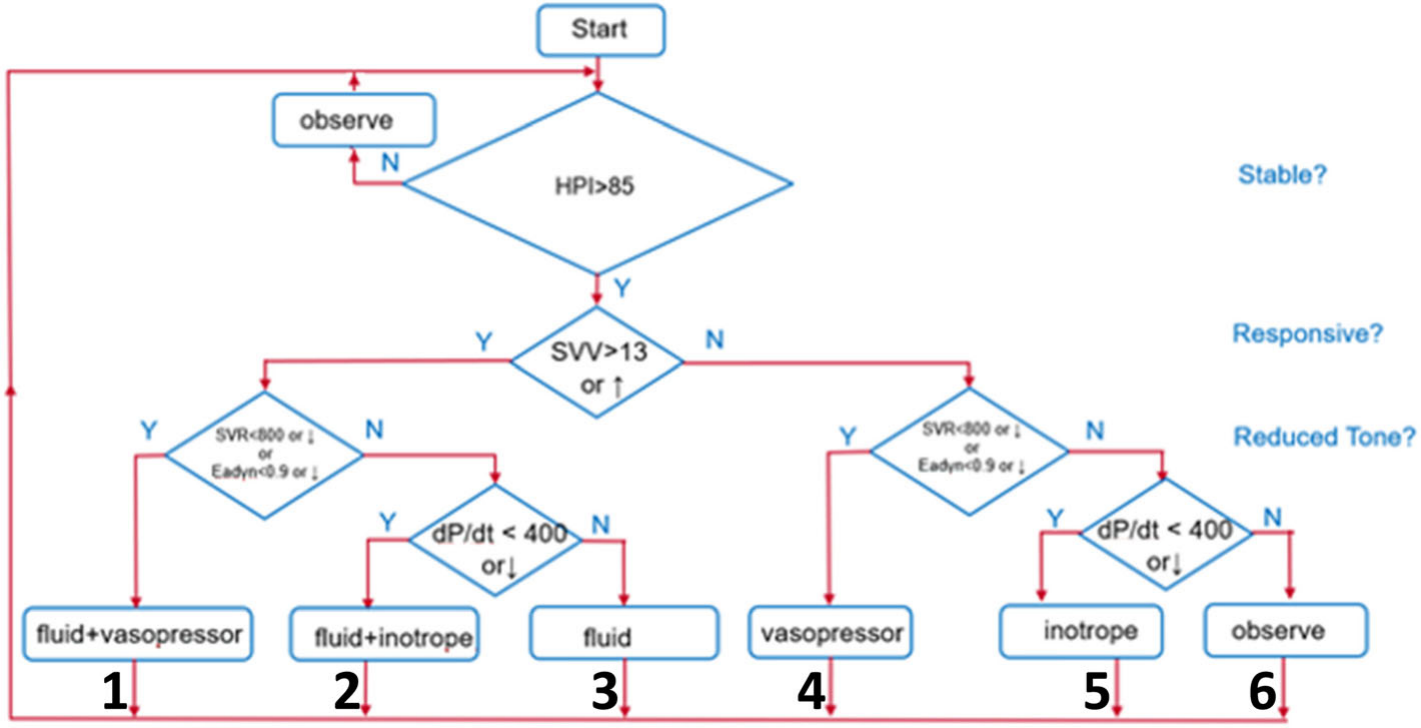
Conceptual framework for the hemodynamic management. *HPI* hypotension prediction index, *SVV* stroke volume variation, *SVR* systemic vascular resistance, *Ea*_*dyn*_ dynamic arterial elastance

In patients assigned to unblinded monitoring, clinicians will additionally have access to HPI index, ranging from 0 to 100, corresponding to the likelihood of hypotension in the coming minutes. The system generates an alert to clinicians when HPI reaches or exceeds 85. To standardize interpretation of hemodynamic values, participating clinicians will agree to the conceptual framework presented in Fig. 3, which considers the possibilities of hypovolemia, vasoplegia, and decreased contractility, and recommend potential interventions. To the extent possible, an in-room investigator will assign the HPI alerts to one of the six potential intervention categories: fluids only, fluids and inotropes, fluids and vasopressor(s), vasopressor(s) only, inotropes only, and observation. The investigator’s conclusion will be shared with clinicians. Clinicians will nonetheless be free to choose any intervention they believe to be appropriate, and the actual intervention will be recorded.

In all cases, good clinical judgment will prevail and the attending anesthesiologist will take all necessary steps to provide optimal and safe care. Blood and blood products will be given per clinical judgment.

Patients will be blinded to group allocation. Anesthesiologists, surgeons, and other intraoperative personnel cannot be blinded to monitoring allocation. However, other investigators and the data-analysis team will remain blinded.

Trial-related management will end when surgery finishes. Patient disposition to a critical care unit or routine surgical ward will be determined by the clinical team per routine.

### Measurements

#### Baseline information

We will record demographic and morphometric characteristics, including height, weight, age, sex, ASA physical status, and self-declared ethnicity. As available, we will collect social history (tobacco and alcohol use), medical history (pulmonary disease, cardiovascular disease, neurologic disease, drug use (including statins, ß blockers, oral hypoglycemic agents, and/or insulin), non-steroidal anti-inflammatory drugs (NSAIDs), diabetes (whether insulin-dependent or not), and glucose-tolerance test results, preoperative hemoglobin and hematocrit, blood urea nitrogen and creatinine, electrolytes, preoperative electrocardiogram, and hemoglobin A_1_c (HbA_1_c).

#### Perioperative data

Intraoperative care data will be accessed from the electronic anesthesia information management system. Anesthetic data will include the volatile anesthetic dose in minimum alveolar concentration-hours (MAC-hours), as well as total doses of propofol, opioids, and sedative hypnotics. Hemodynamic and respiratory values, Bispectral Index, and esophageal temperature will be recorded at regular intervals intraoperatively. Blood loss will be estimated; urine output and fluid administration, including colloids and allergenic blood, will be recorded. Intraoperative use and total dose of vasoactive drugs as well as antibiotic administration will be recorded. If available, arterial blood gas results will be recorded.

#### Blood pressure measurements

Hemodynamic data from the HPI software will be secured on Cleveland Clinic computers for subsequent analysis and manually recorded at 5–15-min intervals. In the unblinded group an investigator will evaluate HPI alerts and document the probable cause based on advanced hemodynamic parameters using the conceptual framework presented in Fig. 3 and the actual intervention.

### Follow-up

Patients will be followed for the major and minor complications shown in Table 1. Blood will be sampled for generation-4 troponin T preoperatively, and on the first and second postoperative days while patients remain hospitalized, which will detect 93% of 30-day myocardial injury [10]. When troponin T elevations are detected, patients will be queried about ischemic symptoms and, when practical, an electrocardiogram and echocardiogram will be obtained. Other study outcomes will be appropriately evaluated and documented. Length of stay will be calculated from the end of surgery to discharge or death (Table 2).

**Table 1:** Outcomes and measurements

	Outcomes	Measurements
1. Primary	TWA MAP drop under 65 mmHg threshold	Intraoperative record from EV1000 monitor, Edwards Lifesciences
2. Secondary	TWA MAP drop under 60 mmHg threshold	Intraoperative record from EV1000 monitor, Edwards Lifesciences
	TWA MAP drop under 55 mmHg threshold	Intraoperative record from EV1000 monitor, Edwards Lifesciences
3. Exploratory	Composite of death, stroke, or MINS	From Cleveland Clinic Perioperative Health Documentation System PHDS and troponin first three postoperative mornings while in hospital from EHR
	Acute kidney injury	AKIN, creatinine
	Quality of recovery (QoR-15)	POD 3, patient interview
	Post-operative morbidity survey (POMS)	POD 3, EHR
	Transfusion requirements (ml)	From PHDS
	Amounts of intraoperative crystalloid and colloid (ml)	From PHDS
	Amount of vasoactive: phenylephrine, ephedrine, nor-epinephrine, epinephrine, dobutamine (mg)	From PHDS
	Advanced hemodynamic variables	From EV100
	Hospital length of stay	From PHDS
	Hospital readmission within 30 days	From EHR

*AKIN* Acute Kidney Injury Network, *EHR* electronic health record, *MAP* mean arterial pressure, *MINS* myocardial injury in non-cardiac surgery, *PHDS* Perioperative health documentation system, *PHDS POD* post-operative day, *TWA* time-weighted average

**Table 2:** Schedule of study events

Data	Screening evaluation	Enrollment	Hemodynamic monitoring	Postoperative day 3
Informed consent	x			
Inclusion/exclusion criteria evaluation	x			
Medical history		x		
Demographic		x		
Vital signs		x	x	
Surgery information		x		
Monitoring duration			x	
Device memory data			x	
Adverse events			x	x
Hemodynamic management information			x	
Intraoperative medications			x	
Quality of recovery				x

### Data analysis

Randomized groups will be compared on baseline variables using the standardized difference (difference in means or proportions divided by the pooled standard deviation), and such variables will be considered balanced if the absolute standardized difference is < 1.96 sqrt (2/N), where N is the per group sample size. Imbalanced variables will be adjusted for in all analyses.

The treatment effect of HPI guidance (versus no HPI guidance) on the primary outcome of intraoperative time-weighted average (TWA) MAP under a threshold of 65 mmHg will be assessed with a Wilcoxon-Mann-Whitney 2-sample two-tailed test, or analogous test to account for imbalanced baseline variables or non-normal distribution of the outcome, as needed. Secondary and exploratory continuous outcomes will be similarly analyzed. We will assess agreement between clinician and decision support flow chart (ordered decisions from 1 to 6; Fig. 3) using weighted Cohen’s kappa, and specifically the Cicchetti-Allison weights [11]. The weighted kappa will accommodate the ordered nature of the rating outcome, giving a weight of 1.0 for perfect agreement, and increasingly smaller weight for larger disagreements.

Randomized groups will be compared on binary outcomes using a Pearson chi-square test or logistic regression to adjust for baseline variables, as needed, and on other outcomes using either the Wilcoxon-Mann-Whitney test or a proportional odds logistic regression model, as appropriate.

For all analyses, focus will be on the estimated treatment effects and their confidence intervals, as well as the *P* values. SAS statistical software, Carey, NC, will be used for all analyses.

### Sample size considerations

Using our institutional database, we observed a mean (standard deviation) area under a MAP < 65 of 80 (127) mmHg.min and median [quartiles] of 24 [1, 121] mmHg.min in a representative sample of patients having non-cardiac surgery (unpublished data). AUC-MAP represents depth and duration of hypotension. AUC-MAP = TWA-MAP × duration of observation. The data were highly skewed, with a non-trivial proportion of zero values. We therefore used a non-parametric test Wilcoxon-Mann-Whitney test to estimate sample size. Based on these data, a sample size of 213 will provide 80% power for detecting an approximately 20% relative reduction in the mean of the primary outcome of AUC-MAP < 65 mmHg.min.

## Discussion

Our goal is to determine whether using HPI software reduces the duration and severity of intraoperative hypotension. The goal is important because intraoperative hypotension is associated with adverse outcomes, including myocardial injury [12], acute kidney injury [6, 12], and 30-day mortality [5, 13]. Furthermore, limited randomized data suggest that hypotension avoidance reduces the risk of postoperative organ failure [8]. To the extent that HPI software reduces hypotension, it may also reduce serious complications.

We will use Flotrac IQ sensors for invasive arterial monitoring because it is necessary for proper functioning of HPI software. In blinded group, we chose not to provide advanced hemodynamic information such as cardiac output because these values are not currently available to our clinicians. Furthermore, HPI is designed to work as a complete hypotension management software and therefore this trial will evaluate both the HPI prediction and the advanced hemodynamic parameters.

HPI and Flotrac IQ are designed to work in conjunction with an arterial catheter. Planned use of an arterial catheter is thus an inclusion criterion, and will restrict enrollment to relatively sick patients having major procedures—but these are also the patients who have the greatest cardiovascular risk. HPI requires a good-quality arterial line waveform. To the extent that the waveform is damped or varies consequent to changes in patient position or other mechanical issues, predictions may be compromised. Our research team will continuously monitor the quality of the arterial waveform and adjust the catheter and system as necessary.

Once hypotension is predicted by the HPI, clinicians will vary in how proactively they respond, and some may poorly comply with our intervention guidelines, which are recommendations rather than requirements. We will allow considerable flexibility on the theory that clinicians will optimize patient safety even though allowing flexibility will add variability to the results. We will, however, record divergences between the algorithm and actual clinical management and plan sensitivity analyses to evaluate the effects of concordant and discordant responses on the extent and duration of hypotension.

**Fig. 4:**
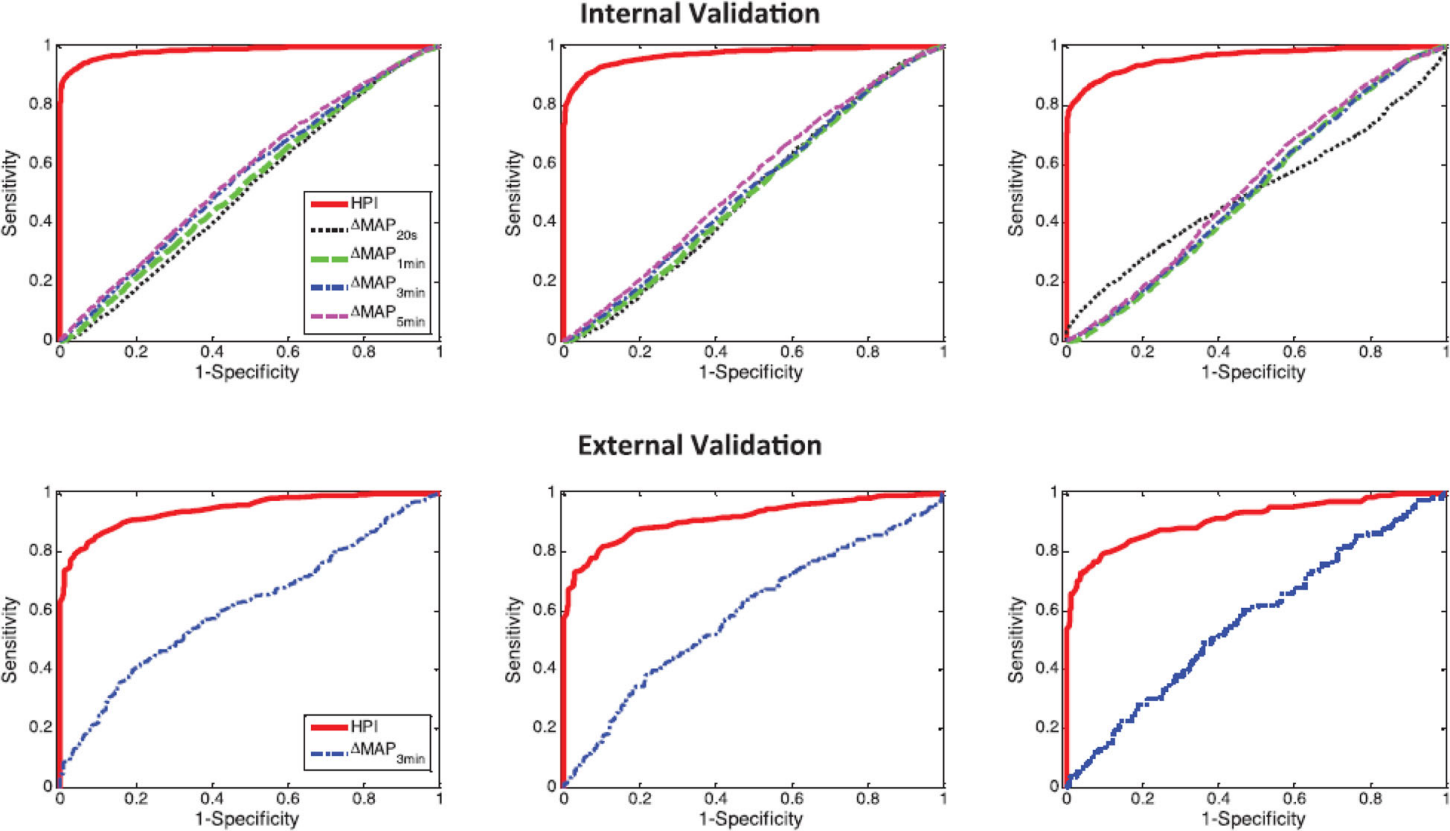
Reliability of the Hypotension Prediction Index (*HPI*) to predict a hypotensive event in time prior to the event [9]. *MAP* mean arterial pressure

HPI software provides reliable prediction of hypotension with good internal and external validation (Fig. 4) [9]. As with many clinical devices, however, HPI software is based on proprietary algorithms and clinicians maybe reluctant to adopt a system that they do not fully understand**.**

## Summary

We plan a randomized trial to determine whether guiding intraoperative hemodynamic management with HPI software reduces the duration and severity of hypotension during non-cardiac surgery. Since hypotension is associated with perioperative complications, patients may benefit if the HPI and associated hemodynamic data reduce the amount of intraoperative hypotension.

## Trial status

Protocol version 1.0; April 2018. Recruitment began July 12, 2018 and will be completed July, 2020.

## Additional file

Additional file 1: SPIRIT 2013 checklist: Recommended items to address in a clinical trial protocol and related documents. (DOC 130 kb)

## Abbreviations

ASA: American Society of Anesthesiologists
HPI: Hypotension Prediction Index
MAP: Mean arterial pressure
SAS: Statistical Analysis System
TWA: Time-weighted average
